# Distance to primary care facilities and healthcare utilization for preschool children in rural northwestern Burkina Faso: results from a surveillance cohort

**DOI:** 10.1186/s12913-021-06226-5

**Published:** 2021-03-09

**Authors:** Catherine E. Oldenburg, Ali Sié, Mamadou Ouattara, Mamadou Bountogo, Valentin Boudo, Idrissa Kouanda, Elodie Lebas, Jessica M. Brogdon, Ying Lin, Fanice Nyatigo, Benjamin F. Arnold, Thomas M. Lietman, for the Étude CHAT Study Group

**Affiliations:** 1 Francis I Proctor Foundation, University of California, 513 Parnassus Ave, Box 0412, San Francisco, CA 94143 USA; 2 Department of Ophthalmology, University of California, San Francisco, USA; 3 Department of Epidemiology & Biostatistics, University of California, San Francisco, USA; 4 Centre de Recherche en Santé de Nouna, Nouna, Burkina Faso

**Keywords:** Primary healthcare, Burkina Faso, Pneumonia, Diarrhea, Malaria, Healthcare utilization

## Abstract

**Background:**

Delays in care-seeking for childhood illness may lead to more severe outcomes. We evaluated whether community distance from a primary healthcare facility was associated with decreased healthcare utilization in a rural district of northwestern Burkina Faso.

**Methods:**

We conducted passive surveillance of all government-run primary healthcare facilities in Nouna District, Burkina Faso from March 1 through May 31, 2020. All healthcare visits for children under 5 years of age were recorded on a standardized form for sick children. We recorded the age, sex, and community of residence of the child as well as any diagnoses and treatments administered. We calculated healthcare utilization per 100 child-months by linking the aggregate number of visits at the community level to the community’s population of children under 5 months per a census that was conducted from August 2019 through February 2020. We calculated the distance between each community and its corresponding healthcare facility and assessed the relationship between distance and the rate of healthcare utilization.

**Results:**

In 226 study communities, 12,676 primary healthcare visits were recorded over the three-month period. The median distance between the community and primary healthcare facility was 5.0 km (IQR 2.6 to 6.9 km), and median number of healthcare visits per 100 child-months at the community level was 6.7 (IQR 3.7 to 12.3). The rate of primary healthcare visits declined with increasing distance from clinic (Spearman’s rho − 0.42, 95% CI − 0.54 to − 0.31, *P* < 0.0001). This relationship was similar for cause-specific clinic visits (including pneumonia, malaria, and diarrhea) and for antibiotic prescriptions.

**Conclusions:**

We documented a distance decay effect between community distance from a primary healthcare facility and the rate of healthcare visits for children under 5. Decreasing distance-related barriers, for example by increasing the number of facilities or targeting outreach to more distant communities, may improve healthcare utilization for young children in similar settings.

**Supplementary Information:**

The online version contains supplementary material available at 10.1186/s12913-021-06226-5.

## Background

Childhood mortality is declining globally, although it is declining at different rates both across and within countries [1, 2]. For example, mortality rates tend to be higher in rural compared to urban areas, and within urban areas in informal settlement areas compared to more developed neighborhoods in cities [1, 3, 4]. The majority of post-neonatal childhood mortality in sub-Saharan Africa is infectious. Major drivers of persistently high childhood mortality are structural, including extreme poverty and lack of access to healthcare. In areas with declining childhood mortality, deaths often occur at home [5]. Barriers to accessing healthcare may therefore result in sub-populations with increased risk of childhood mortality, even in settings where overall mortality rates are declining.

In Burkina Faso, healthcare for children under 5 years of age has been provided by the Ministry of Health free of charge since 2016 [6]. Prior to introduction of free care, user fees for healthcare services had been described as a barrier to accessing care [7]. However, substantial barriers to care likely remain. Children with reduced access to primary care may be at increased vulnerability to adverse health outcomes including mortality and thus may be a priority population for targeting interventions for mitigating child mortality and morbidity. We assessed the relationship between primary healthcare utilization among children under 5 years of age (defined as the number of clinic visits per population) and distance to the primary healthcare facility to assess whether physical distance from a facility reduces healthcare-seeking behavior in rural Burkina Faso. We hypothesized community-level healthcare utilization would be decreased for communities with increased distance to the healthcare facility. We additionally evaluated whether increasing distance was correlated with antibiotic prescription practices because antibiotic prescribing may be more dependent on clinician prescribing practices rather than healthcare seeking behaviors. We hypothesized that antibiotic prescription rates would be higher in communities closer to healthcare facilities, which could have implications for antimicrobial resistance selection [8].

## Methods

### Study setting

This study took place in Nouna district in northwestern Burkina Faso [9]. The area is primarily rural and agrarian [10]. All communities in Nouna District and all primary healthcare facilities were eligible for inclusion in the study [9]. Data on clinic visits were collected from March 1 through May 31, 2020, which corresponds to the dry season and low malaria transmission season in the study area, and the period in between the annual harvest and the annual planting season. Data were collected during the dry season due to logistical reasons from the parent study so that timing was shortly after the baseline census (described below) [9]. Primary healthcare in rural Burkina Faso is delivered via *Centres de Santé et de Promotion Sociale* (CSPS), which are typically nurse-led rural healthcare units that deliver first-line preventive and therapeutic care. These represent the first level of the healthcare system in Burkina Faso, and cases requiring more advanced care are referred to district-level hospitals. Each CSPS serves several communities in a given catchment area that is defined by the government.

### Ethical approval

This study was reviewed and approved by the Comité National d’Ethique pour la Recherche (National Ethics Committee of Burkina Faso) in Ouagadougou, Burkina Faso and the Institutional Review Board at the University of California, San Francisco in San Francisco, California. Verbal informed consent was obtained from the head of each household for participation in the census, and written informed consent was obtained from the caregiver of each child included in the study.

### Census

We utilized the baseline census from the Community Health with Azithromycin Trial to estimate the population of children under 5 years of age as the denominator for calculations of healthcare visits per population. The census took place from August 2019 through February 2020. Complete methods for the census have been previously reported [9]. The head of each household in each study community was asked if there were any children under 5 years of age living in the household, and those in households with children were asked to list each child. The date of birth and sex of each child under five was recorded in the census and the household’s Global Positioning System (GPS) coordinates were recorded.

### Primary healthcare surveillance

We recorded each sick child encounter for all children under 5 years of age at each primary healthcare facility in the study area. Well-child visits (e.g., routine post-natal visits or vaccination visits) were not recorded. Follow-up visits were excluded, and thus data represent only the first visit for each case of illness. Data were extracted from ledgers issued by the Ministry of Health for recording clinic visits and entered into an electronic form. Information extracted included the date of the visit, the child’s age, sex, community of residence, diagnosis, and any prescribed treatments. GPS coordinates were recorded for each health facility in the study area.

### Statistical methods

To calculate the number of primary health clinic encounters per population, we utilized data from the census to estimate denominators and summed the number of clinic visits for each individual community. We considered the assigned healthcare facility to be the healthcare facility assigned to the community by the government of Burkina Faso. We assessed the assumption that most care was sought at the facility assigned by the government by assessing the community of residence for all sick child visits. Some study communities more often used healthcare facilities that were closer to them than their government-assigned healthcare facility (*N* = 8 communities; 3.5%). When this occurred for more than 50% of child visits from that community, we assigned the study community to the healthcare facility they actually used.

We estimated the number of clinic visits per 100 children per month over the three-month period of observation by dividing the number of visits per month per community to their linked healthcare facility (either assigned by the government or the one most commonly used by the community as previously described) by the total population per the census and multiplying by 100. We then calculated diagnosis-specific visits, including malaria, pneumonia, and diarrhea which are the three leading causes of child mortality in the study area. We then evaluated the rate of antibiotic prescription by calculating the total number of antibiotic prescriptions separately and calculation prescriptions per 100 children per month. To estimate the distance from the study community to the healthcare facility used by that community, we took the average of the GPS coordinates from each household in a given study community and calculated the geodetic distance between the study community and the health facility using the *geodist* function in Stata 15.1 (StataCorp, College Station, TX). Data were analyzed descriptively using scatterplots and LOESS curves and Spearman rank correlation coefficients were calculated to assess the community-level association between healthcare clinic usage and distance from the community to the primary healthcare clinic. Spearman rank correlation coefficients were used due to the non-linear relationship between distance measures and healthcare utilization [11]. We then constructed regression models adjusting for the total population of children under 5 years of age in the community to assess if any relationship between healthcare utilization and distance from the community to the healthcare facility was explained by differences in the size of the community. These models included a quadratic term for distance to account for the non-linear relationship between distance measures and healthcare utilization, and the polynomial fit lines from the regression models were superimposed onto scatterplots. All analyses were conducted in R version 3.6.1 (The R Foundation for Statistical Computing, Vienna, Austria).

## Results

Between March 1 and May 31, 2020, we recorded 16,242 clinic visits at 45 primary healthcare facilities in Nouna District, Burkina Faso. Of these, 12,676 (78.0%) were from children residing in one of 226 study communities and were included in this analysis (Table 1). A total of 44,064 children under 5 years of age were recorded on the census, and communities had a median of 151 children (interquartile range, IQR, 86 to 246 children). The median distance between the community and primary healthcare facility was 5.0 km (IQR 2.6 to 6.9 km). The median number of healthcare visits per 100 child-months at the community level was 6.7 (IQR 3.7 to 12.3).

**Table 1:** Descriptive characteristics of study sample

*Population-based characteristics*	
Communities	226
Total children	44,064
Age group	
0 to 6 months	3450 (7.8%)
7 to 12 months	4229 (9.6%)
13 to 18 months	4712 (10.7%)
19 to 24 months	4464 (10.1%)
25 to 36 months	9337 (21.2%)
37 to 48 months	9178 (20.8%)
49 to 60 months	8694 (19.7%)
*Clinic visits (March through May 2020)*	
Primary healthcare facilities	45
Total visits	12,676
Age group	
0 to 6 months	2859 (22.6%)
7 to 12 months	2884 (22.8%)
13 to 18 months	1953 (15.4%)
19 to 24 months	1554 (12.3%)
25 to 36 months	1835 (14.5%)
37 to 48 months	969 (7.6%)
49 to 60 months	622 (4.9%)
Reason for visit	
Pneumonia	4754 (37.5%)
Malaria	3181 (25.1%)
Diarrhea	1157 (9.1%)
Other diagnoses	3584 (28.3%)
Total antibiotic prescriptions	7635
Antibiotic class	
Amoxicillin	5343 (70.0%)
Cotrimoxazole	706 (9.2%)
Erythromycin	808 (10.6%)
Other classes	778 (10.2%)

Among children attending a healthcare visit, the median age was 14 months (IQR 7 to 26 months) and 43.8% were female. The rate of healthcare visits per 100 child-months was higher for younger children and decreased steadily among older children, from 27.6 visits per 100 child-months among children < 6 months of age to 2.4 visits per 100 child-months among children > 48 to 60 months of age (Supplemental Fig. 1A). The most common diagnoses were pneumonia (37.5% of visits), malaria (25.1% of visits), and diarrhea (9.1% of visits).

Of the 12,676 healthcare visits, an antibiotic was prescribed in 7635 visits (60.2%). Similar to overall healthcare visits, the rate of antibiotic prescription decreased steadily as age increased, from 17.8 antibiotic prescriptions per 100 child-months among children < 6 months of age to 1.4 antibiotic prescriptions per 100 child-months among children > 48 to 60 months of age (Supplemental Fig. 1B). Amoxicillin was the most commonly prescribed antibiotic (70.0% of antibiotic prescriptions), followed by erythromycin (10.6% of antibiotic prescriptions) and cotrimoxazole (9.2% of antibiotic prescriptions).

**Fig. 1:**
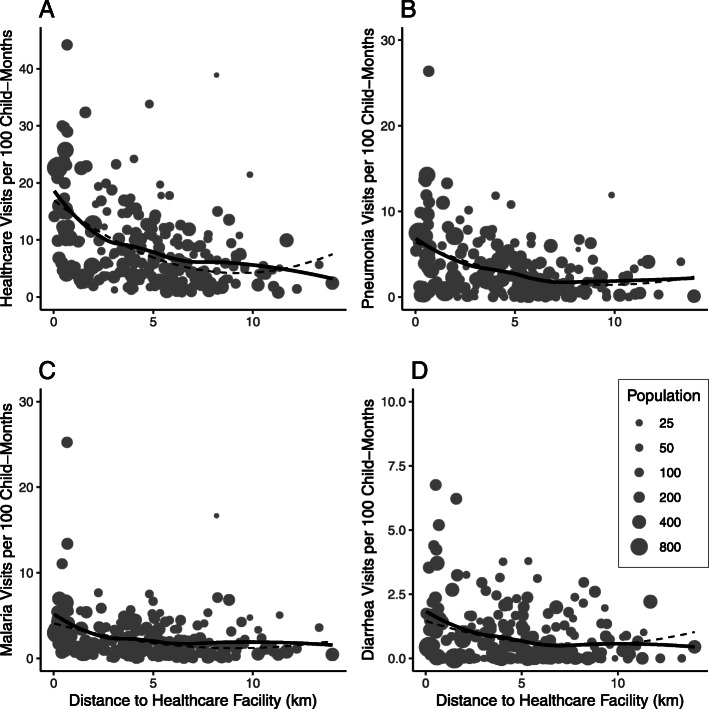
Community-level rate of overall (**a**) and cause-specific (**b**-**d**) clinic visits per 100 child-months by distance in kilometers from the community to the primary healthcare facility. Specific diagnoses include pneumonia (**b**), uncomplicated malaria (**c**), and non-bloody diarrhea (**d**). Grey dots indicate individual communities sized by the community’s population of children aged 0–59 months based on the most recent census. Solid black lines indicate a loess curve and dashed black lines indicate a polynomial fit curve from a quadratic regression model

**Fig. 2:**
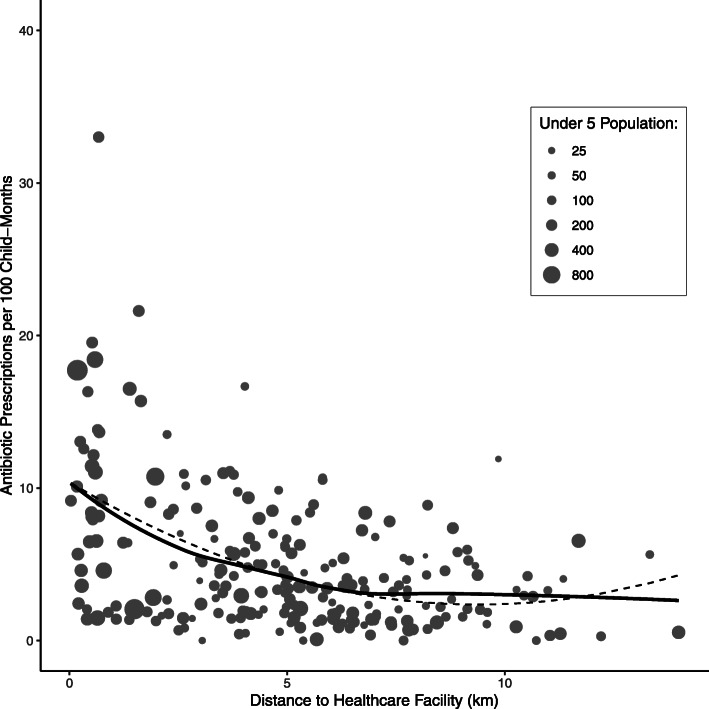
Community-level rate of antibiotic prescriptions per 100 child-months by distance in kilometers from the community to the primary healthcare facility. Grey dots indicate individual communities sized by the community’s population of children aged 0–59 months based on the most recent census. Solid black lines indicate a loess curve and dashed black lines indicate a polynomial fit curve from a quadratic regression model

The total number of community-level visits per 100 child-months declined as distance from the community to the healthcare facility increased (Fig. 1a). The rate of primary healthcare visits declined with increasing distance from clinic (Spearman’s rho − 0.42, 95% CI − 0.54 to − 0.31, *P* < 0.0001). The relationship attenuated with decreasing cause-specific frequency, with the strongest relationship between visits for pneumonia and distance to the healthcare facility (Spearman’s rho − 0.33, 95% CI − 0.44 to − 0.21, *P* < 0.0001; Fig. 1b), followed by malaria (Spearman’s rho − 0.31, 95% CI − 0.42 to − 0.18, *P* < 0.0001; Fig. 1c), and diarrhea (Spearman’s rho − 0.24, 95% CI − 0.36 to − 0.11, *P* = 0.0003; Fig. 1d). The trend for antibiotic prescription (Fig. 2) was similar to that for overall healthcare visits (Spearman’s rho − 0.38, 95% CI − 0.49 to − 0.26, *P* < 0.0001). The proportion of visits in which an antibiotic was prescribed did not vary by distance to the clinic (Spearman’s rho = 0.04, 95% CI − 0.09 to 0.17, *P* = 0.55). Polynomial regression models including a quadratic term for distance from the community of residence to the healthcare facility and adjusting for the total population of children under 5 years of age found similar results for primary healthcare visits and antibiotic prescriptions, as shown in Figs. 1 and 2.

## Discussion

Utilization of primary healthcare was common for children under 5 years of age in this rural region of Burkina Faso, and utilization decreased as distance between the primary healthcare facility and the community of residence. This relationship persisted for care-seeking for the three major causes of child mortality in the region (pneumonia, malaria, and diarrhea), although it was attenuated. Early care-seeking for pneumonia, malaria, and diarrhea is important to prevent mortality from these conditions [12]. Ability to rapidly seek care for childhood illness includes both caregiver recognition of a child’s illness as well as overcoming structural barriers to care-seeking. Extended distance from a community of residence to the primary healthcare facility may represent a structural barrier to seeking care. Although the removal of fees for children under 5 years of age overcomes some financial barriers to accessing healthcare for children, extended travel times likely impose additional time and financial barriers to accessing care and caregivers may delay care-seeking. These results reflect those of previous studies in sub-Saharan Africa, which have demonstrated a distance decay effect in the healthcare utilization for children with increasing distance to healthcare facilities [13, 14]. Taken together, these results indicate that distance to a healthcare facility is a structural barrier to care-seeking, and reducing these barriers is a priority for improving child health outcomes.

Similar to other studies [13, 15], the sharpest decline in clinic utilization happened at smaller distances, with the sharpest decline in utilization occurring before 5 km of distance between the health facility and the community of residence. A sharper decay may be observed at shorter distances because caregivers who live very close to healthcare facilities may be more likely to visit for minor ailments compared to those who live further away. Commonly used transportation modalities in the study area include personally owned motorcycles and bicycles and by foot. Major roadways in the study area are tarmac, but most roadways are not paved. Travel times likely vary both by quality of the road and transport mode. We did not collect data on time for travel between each community and healthcare facilities, which may be affected by quality and location of roads. Although geographic distance is correlated with travel time, it may not capture the entire relationship between healthcare use and physical locations of residences and healthcare facilities, which may include factors such as transit time or available transportation modes. Future studies could additionally measure travel times, which may provide additional insight into distance-related barriers to care seeking.

The relationship between cause-specific healthcare visits and distance did not substantially vary for the three major causes of childhood mortality, including pneumonia, malaria, and diarrhea. Correlations were attenuated with decreasing frequency of each diagnosis. Assuming that the true incidence of each condition does not differ for children closer compared to further from a healthcare facility, the persistence of this relationship across diagnoses that can have severe outcomes suggests that care is not sought for all children who may benefit. Although we did not have data on severity or outcomes for children seeking care for these diagnoses, it is possible that care is sought later for children who live further from healthcare facilities, due to increased burden in accessing care. Early symptoms such as fever may be perceived as mild and not require care [16, 17]. If care is sought later for children they may be more likely to develop severe disease and have worse outcomes. Reducing transit time or increasing the number of healthcare facilities (and thus decrease distance required to travel to facilities) may improve outcomes for children if severity of illness is a factor in the decision to access care for children.

Nearly two-thirds of healthcare visits resulted in an antibiotic prescription, and the rate of antibiotic prescription per visit did not change significantly with increasing distance to healthcare facilities. Although it is possible that caregivers could access antibiotics outside of the public healthcare system that would be missed by CSPS surveillance, previous work in this study area demonstrated that most childhood antibiotic distribution is via public facilities and thus would be recorded in this system [18]. Antibiotic use is known to select for antibiotic resistance in both individuals and populations, and this may have implications for the epidemiology of antibiotic resistance in the area [8, 19, 20]. If community-level use of antibiotics is predictive of resistance patterns, communities further from healthcare facilities may have reduced prevalence of antibiotic resistance.

The results of this study should be considered in the context of several limitations. As previously mentioned, we did not collect data from private facilities or hospitals, and only included healthcare visits that occurred at government-run primary healthcare clinics. If care was sought via other sources that were not captured as part of our surveillance and these sources differed in geographic distribution, it could affect the association between distance from the healthcare facility and healthcare utilization. However, the vast majority of healthcare in the study area is distributed free of charge via the government system. We were not able to link individual children seeking care at healthcare facilities to individual households in the census; we were only able to link them to their community of residence. Especially in larger communities there may be variation in household location within community that may lead to differences in healthcare utilization patterns. We did not collect data on well-child visits, such as vaccination visits, and thus cannot comment on whether healthcare utilization patterns differ for preventative care. If such a trend persisted in accessing preventative care, this could have important implications for the success of vaccination and other prevention programs. Data were collected only during the dry season. Given the highly seasonal nature of malaria in the study area, trends may be different during the rainy season and patient volumes may be increased, which could affect these relationships. These results may not be generalizable to the rainy season.

## Conclusions

In this rural district of northwestern Burkina Faso, we found evidence of a distance decay effect in healthcare seeking for children at government-run primary healthcare facilities. This effect persisted across multiple diagnoses and for antibiotic use. Increasing access to primary healthcare facilities may facilitate timely care-seeking for children, which may improve outcomes from common causes of childhood mortality. Increasing the number of healthcare facilities may decrease distance to the healthcare facility and improve care utilization. Other interventions that deliver care directly to communities, such as working with community health workers, may be helpful for improving child health outcomes.

## Supplementary Information

**Additional file 1: Supplemental Fig. 1.** Clinic visits per 100 child-months by age group (A), overall (black line) and cause-specific visits including pneumonia (yellow line), malaria (red line), and diarrhea (green), and antibiotic prescriptions per 100 child-months (B) overall (black line) and by antibiotic, including amoxicillin (red line), erythromycin (green line), and cotrimoxazole (purple line). Amoxicillin includes amoxicillin only, amoxicillin-clavulanic acid, and penicillin.

## Data Availability

Datasets analyzed during the current study are available from the corresponding author upon reasonable request.

## Abbreviations

CI: Confidence interval
CSPS: Centre de santé et de promotion sociale
GPS: Global positioning system
IQR: Interquartile range
